# Detection of Ludic Patterns in Two Triadic Motor Games and Differences in Decision Complexity

**DOI:** 10.3389/fpsyg.2017.02259

**Published:** 2018-01-05

**Authors:** Miguel Pic Aguilar, Vicente Navarro-Adelantado, Gudberg K. Jonsson

**Affiliations:** ^1^Consejeria Educacion Canarias, University of La Laguna, San Cristóbal de La Laguna, Spain; ^2^Human Behavior Laboratory, University of Iceland, Reykjavik, Iceland

**Keywords:** Theme, triad, motor game, structure, T-patterns

## Abstract

The triad is a particular structure in which an ambivalent social relationship takes place. This work is focused on the search of behavioral regularities in the practice of motor games in triad, which is a little known field. For the detection of behavioral patterns not visible to the naked eye, we use Theme. A chasing games model was followed, with rules, and in two different structures (A↔B↔C↔A and A → B → C → A) on four class groups (two for each structure), for a total of 84, 12, and 13 year old secondary school students, 37 girls (44%) and 47 boys (56%). The aim was to examine if the players' behavior, in relation to the triad structure, matches with any ludic behavior patterns. An observational methodology was applied, with a nomothetic, punctual and multidimensional design. The intra and inter-evaluative correlation coefficients and the generalizability theory ensured the quality of the data. A mixed behavioral role system was used (four criteria and 15 categories), and the pattern detection software Theme was applied to detect temporal regularities in the order of event occurrences. The results show that time location of motor responses in triad games was not random. In the “maze” game we detected more complex ludic patterns than the “three fields” game, which might be explained by means of structural determinants such as circulation. This research points out the decisional complexity in motor games, and it confirms the differences among triads from the point of view of motor communication.

## Introduction

Motor games with rules enclose players behavior regularity due to the expectations of the roles. The regularities of these behaviors also depend on the communication structure to which the players are subjected. Play behaviors are an orderly way of communicating in games with rules. The relationships between the roles of the games and its time are two key aspects of the analysis of the relationships showed by the players, in this way triadic relationships are defined. Triad motor games are composed of three decisional units (three individuals or three groups of individuals), in a context of motor communication (Parlebas, 1981), with players' autonomy to act according to strategic interests (Pic and Navarro, 2017).

To speak about regularity of behavior in games is to talk about the outcome of a logic of each motor situation (Parlebas, 1981, 1988, 2005a,b,c). This logic has been developed by Parlebas' motor praxiology, and studied in sports games. However, it is necessary to reach a deeper level of analysis of issues that are hidden from the chaining of roles in different games. Knowing the time regularity of the motor sequences used by the players provides more information about the background of the transcendent playing relations. Linking the role to the time requirement implies ensuring a more precise, consistent, and revealing pattern.

Motor games show varying levels of complexity; one of these complexities is provided by the triad game (Parlebas, 2011). The inclusion of a third triadic element incorporates coalliance (Gamson, 1961) into the playful scenario (Navarro and Pic, 2016) increasing the habitual complexity of the dual game. In general, games have a consubstantial random component that comes from their degree of uncertainty but, in the face of this, players struggle to control that uncertainty and to approach their strategy. Triad motor games have in complexity a research challenge, because they delve into cooperation (Pic and Navarro, 2017) even though players may be adversaries.

Complexity in triadic games remains a research challenge. Complexity studies have indicated the importance of including the time scale in research (Balagué et al., 2013). In triad, property activation is relevant and susceptible to becoming constraints (Davids et al., 2008). To analyze complexity, three deepening levels are available: (a) the structure of the game, (b) the role and (c) the observed behavior. The complexity of the triad structures (Simmel, 1950; Caplow, 1956, 1959; Wasserman, 1975; Wasserman and Faust, 2013) refers to the amount of relationships established between the teams in the game, and the decisional range of the roles of a game brings with it a series of possible relationships with other roles. Finally, the “strategic whole” is therefore unsurpassable and, through the role, the behaviors put into practice by the players must be made operative in order to unravel the true complexity.

In dual games, the identity of each party is reflected in the antagonism of their relationships. Nevertheless, in the triadic census (Moody, 1998), based on the relation typology, a great variety of connections is verified, which could activate certain properties. Two properties such as reciprocity and circulation affect structures 1 (A↔B↔C↔A) and structure 2 (A → B → C → A) in a different way.

Reciprocity is understood as the two-way communication connection between two teams (A↔B), while circulation is the sequence of connections from one source to the same starting point, completing one cycle. Structure 2 does not have reciprocity, but with one-way circulation, however, structure 1 has 3 reciprocities and two-way circulation, that is, it has two origins. Their reciprocity is affected in the confrontation between the three teams, because each duel would annul itself reciprocally. In relation to the circulation property in structure 2, in simultaneous capture games, if the players of team A take prisoners of the players of team B, it means that most of the players of team C will remain as free players because there are scarce possibilities for team B players to get some catch from team C. That is, team A, following the rules of the game and fulfilling their pretensions, hinder their chances of winning the game. These situations, scarcely studied in the motor game, were already analyzed in depth with a social approach by Caplow (1959). It should be noted that we understand complexity as the set of elements, relationships and emergent properties based on a strategic sense. Therefore, in structure 1, there would be greater complexity and average probabilities for the paradox to appear, while in structure 2 the complexity would be less, but the structural paradox would appear due to the relational disposition of its elements.

The internal logic of the motor game leads to focus attention on the role as it is a route of play when players act. Role is a structural indicator that helps to operationalize the motor game, as well as to order the strategic procedures that the players put into practice to reach their objectives. In an apparently uncontrolled context in a game, each driving situation is unique and unrepeatable. Role represents the path, the structure of communication support, and T-patterns (detected with Theme) emerge from the observed behaviors. In this sense, the T-patterns found are not rules of decision for the game, but seemingly random events which are timely and behaviorally organized, though. For this reason, an ecological methodology (Anguera and Hernández-Mendo, 2014) is required to advance in the study of these structures based on the motor interaction of three teams, as well as the inclusion of the time parameter (Magnusson, 2000). To find out T-patterns, the observation of games offers methodological advantages with which to reduce the complexity of the triadic game.

In the observational methodology (Lapresa et al., 2013a; Anguera and Hernández-Mendo, 2014, 2016) the use of analysis techniques that help to evidence the construction of behavioral structures with time regularity has been increasing. As it is well known, THEME is a software (Magnusson, 1996, 2000) that detects T-patterns (Borrie et al., 2002; Jonsson et al., 2006; Casarrubea et al., 2015) by combining ordered events which occur at relatively invariable time distances.

Motor games have not been studied using the T-pattern algorithm before, despite their communicational richness, but sports have. There are differences of a time sense between the first and second part in soccer matches in high level competitions (Cavalera et al., 2015) detected by using an observational methodology and THEME (Magnusson, 1996, 2000). T-patterns have been identified in the motor interactions of F.C. Barcelona (Camerino et al., 2012) and finalized strikes in goal in futsal (Lapresa et al., 2015). In basketball, the effectiveness of offensive play (Fernandez et al., 2009; Lapresa et al., 2013a) and foot position were studied, among other criteria, to try to optimize pitch (Garzón et al., 2011), and it was also studied in combat sports (Camerino et al., 2014).

Justification of the hypothesis: the decisions of a triadic motor game players show the complexity of game structures. Strategic regularity represents a degree of strategic organization which players put into practice when playing. The detection of T-patterns is evidence of the logic of strategic sense, ordered in roles. The decisional chance is greater the less able the players are to carry out actions that allow obtaining advantages. When confronting two triadic motor games with different distribution of directed graphs (communication flows), two communication conditions are tested when developing the game strategy; the game “the maze” is more complex due to the number of reciprocities, while “the three fields” does not have any reciprocity. In addition, the one-way communication of the game “the three fields,” directly activates a structural paradox with consequences on the strategy of teammates and/or rivals.

In this line of research, aimed at the motor game, the objective was to look for temporary regularities in two triad games, under two different communication structures, through game roles and their observable behaviors in practice. For this study, the following generic and specific objectives were considered, respectively.

- Identifying systemic properties in two triadic games and analyzing their influence in practice.
- Discriminating the scope of triadic behavior according to the roles of the game.

Hypothesis:

- The increase in decisional complexity and strategic regularity (T-patterns) is greater in favor of the triadic game with a greater number of reciprocities in its communication.

## Methods

### Design

An observational methodology design was selected for the study. It is a relatively recent methodological approach (Anguera and Hernández-Mendo, 2014, 2016) with application to sports and physical education (Fernández et al., 2012; Hernández-Mendo and Planchuelo, 2013). Specifically, a N/P/M design (Blanco-Villaseñor et al., 2003; Anguera et al., 2011) is applied: (a) Nomothetic (N) because the motor behaviors of different players were recorded; (b) Punctual (P), because the registered games were raised in a precise moment; and finally, (c) Multidimensional (M), since different dimensions (observational criteria) constituting the observation tool were taken into account.

### Participants

The number of players was 84, consisting of 37 (44%) girls and 47 (56%) boys between 12 and 13 years old (M = 12.5; DT = 1) from two secondary schools in the Canary Islands (Spain). The two institutes were located in different cities and islands. The cities were middle-class urban places. Both the groups and the institutes were selected according to accessibility and intentionality (Anguera et al., 1995). The students played two motor games, distributed in 4 class groups; in each center there were two groups (group A, group B) for each game (game 1: Maze and game 2: Three fields). The first game (maze, modified), in both secondary schools, was played by 21 Players and distributed into 7 participants per team (group 1). The second game (Three fields) was practiced with an identical distribution of players in both secondary schools. This study was carried out in accordance with the recommendations of *ethics committee for research and animal welfare* of the University of La Laguna (Spain) with written informed consent from all parents or legals tutors of all participants (Declaration of Helsinki).

### Materials

The two triadic games analyzed were “the maze” (modified) and “the three fields” (modification), which are both chase games. In “the maze” (A↔B↔C↔A), three teams are formed, with the same strengths, and all players try to capture each other simultaneously, under action conditions that regulate only one part of the body for contact. The captured player assumes the role “prisoner,” remaining crouched in the place where he/she was captured; “prisoners” can be released if they are saved by a free player, under the role of “savior” (Navarro, 1995). The team that first turns all adversaries into “prisoners” wins. It is an ambivalent and stable motor communication network (Parlebas, 1988). In the game “the three fields” (modified) (A → B → C → A), the chase cycle between the teams is regulated.

To analyze these games, the motor interactions of the roles were taken as a reference (Table 1). In addition, four indicators were used: roles, group interaction (intragroup, intergroup), communication (emission, reception), and valence (positive and negative; Heider, 1946). All interactions are computed taking into account the vertex or node representing each team (A, B, C) and their corresponding emissions (positive or negative) and receptions (positive or negative), giving rise to three values. In Table 1 and for game 2, the rating (3,3,3) in the “catcher” role as negative emission means that 3 is the value of the negative emission flux of A on the roles of team's B and C, specifically with 1 reception for each catcher, dodger, and savior. And all this happens as intergroup interaction.

**Table 1:** Motor interactions of “the maze” game (modified) and “the three fields” (modified), following the indicators “role,” “game group,” “valence,” and “communication.”

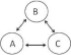		**Intragroup**				**Intergroup**			
		**Emissions**		**Receptions**		**Emissions**		**Receptions**	
		**+**	**−**	**+**	**−**	**+**	**−**	**+**	**−**
Game	Roles								
The Maze	Capture	0,0,0	0,0,0	0,0,0	0,0,0	0,0,0	6,6,6	0,0,0	2,2,2
	Dodger	0,0,0	0,0,0	0,0,0	0,0,0	0,0,0	0,0,0	0,0,0	2,2,2
	Prisioner	0,0,0	0,0,0	1,1,1	0,0,0	0,0,0	0,0,0	0,0,0	0,0,0
	Savior	1,1,1	0,0,0	0,0,0	0,0,0	0,0,0	0,0,0	0,0,0	2,2,2
	Total	1,1,1	0,0,0	1,1,1	0,0,0	0,0,0	6,6,6	0,0,0	6,6,6
		6 intragroup interactions (6 positive, 0 negative) In total (A, B, C = 3 positive emissions and 3 positive receptions)				36 intergroup interactions (0 positive and 36 negative) In total (A, B, C = 18 negative emissions and 18 negative receptions)			
	Total: 42 motor interactions (6 positive and 36 negative: 1 to 6 in favor of rivalry over solidarity)								

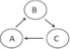		**Intragroup**				**Intergroup**			
		**Receptions**		**Emissions**		**Receptions**		**Emissions**	
		+	−	+	−	+	−	+	−
Game	Roles								
The three fields	Capture	0,0,0	0,0,0	0,0,0	0,0,0	0,0,0	3,3,3	0,0,0	1,1,1
	Dodger	0,0,0	0,0,0	0,0,0	0,0,0	0,0,0	0,0,0	0,0,0	1,1,1
	Prisioner	0,0,0	0,0,0	1,1,1	0,0,0	0,0,0	0,0,0	0,0,0	0,0,0
	Savior	1,1,1	0,0,0	0,0,0	0,0,0	0,0,0	0,0,0	0,0,0	1,1,1
	Total	1,1,1	0,0,0	1,1,1	0,0,0	0,0,0	3,3,3	0,0,0	3,3,3
		6 intragroup interactions (6 positive, 0 negative) In total (A, B, C = 3 positive emissions and 3 positive receptions)				18 Intergroup Interactions (0 positive and 18 negative) In total (A, B, C = 9 negative emissions and 9 negative receptions; 0 positive emissions and 0 positive receptions)			
	Total: 24 motor interactions (6 positive and 18 negative: 1 to 3, in disequilibrium in favor of rivalry over solidarity)								

“The maze” (modified) has a total of 42 motor interactions (6 positive and 36 negative: 1 to 6, in disequilibrium in favor of rivalry). That is, for each cooperative interaction, six antagonists were found. In contrast, when quantifying the motor interaction in “the three fields” game (modified) relational values decrease to 24 motor interactions (6 positive and 18 negative: 1 to 3, in imbalance in favor of rivalry over solidarity). Therefore, a structural difference between these two games comes from doubling antagonism over cooperation. Consequently, these are two games structures with marked differences.

### Observational record system

For the construction of the registration tool, a previous exploratory study (Pic and Navarro, 2014) was used, detecting which strategic options were most demanded by players. From these options, nesting's on the praxiological core “role” were identified. The valence variable was not included in the registration tool because the behavior implies a positive valence (cooperative behavior), a negative valence (rivalry behavior) or an ambivalent behavior (valence positive and negative behavior).

An “*ad-hoc*” tool was used (Table 2) to record the behaviors carried out by boys and girls in triad motor games. The categories are exhaustive and mutually exclusive, merging the multidimensionality of the field format and the referent of the category system.

**Table 2:** Registration system (4 criteria and 15 categories nested in the criteria).

**Criteria**	**Categorie**	**Description**
Catcher (C)	CA	Catches an opponent
Catcher (C)	PA	Chases an opponent
Catcher (C)	DEF	Defends a prisoner
Catcher (C)	P	Passivity
Catcher (C)	ALZAAC	Alliance with adversary
Dodger (E)	EA	Dodges an opponent
Dodger (E)	HA	Runs away from an opponent
Dodger (E)	AC	Helps a fellow escape
Dodger (E)	DLL	Moves to free places
Dodger (E)	NR	Does not recognize being caught
Dodger (E)	ALZAE	Alliance between dodging adversaries
Prisioner (P)	A	In atention
Prisioner (P)	CE	Changes space to make it easier to release
Liberator (L)	TUFC	Touches a prisoner (fellow prisoners)
Liberator (L)	TUFA	Touches a prisoner (adversary prisoners)

The catcher role (C) is identified because it bears the initiative of the game, and it is observable through effective captures (CA) or pursuit actions (PA), which brings together at least two players involved with negative emissions. Sometimes, (C) tries to defend players who are already prisoners and it is then when (DEF) takes place, while passive was the player who was not involved in the game (P). When it was observed that a player belonging to different teams pursued an opponent player, it was considered (ALZAAC), being an ambivalent behavior. Although the previous role had more initiative, the “dodger” role (E) offered a reciprocal response to the catch action (C). In this sense, the dodger (E) will act by dodging on the opponent (EA) but he/she can also flee (HA) or move to free places (DLL). When it is observed that a fellow player intends to interfere in a chase between a catcher and a fleeing player, it was considered (AC). When the action is developed cooperatively between players (ambivalence behaviors) of different teams, it was considered (ALZAE). If a player was caught and did not admit it, this conduct was considered (NR). The prisoner role (P) occurs when a player has been previously captured. If the player is simply standing without facilitating his/her release we say that he/she is not in attention (A), but other times the player facilitates the saving action (CE). The fourth role, liberator (L), tries to save fellow prisoner players (TUFC) but sometimes the release was verified between players of different teams (TUFA).

### Procedure

The images, video recordings, were taken in the educational centers of each group. In order to have a clear observability of each record (Anguera, 2003) and to have at least two recordings of each sequence, inclusion criteria were used. The consent of the study participants and parents/guardians was obtained. The collection of images was made through long distance recordings, making impossible the recognition of the faces of the players in those images. Although these games are usually longer, the first 3 min of each game carried out by each class group were selected. During each practical experience, the spatial conditions were identical, with a play space of 20 × 20 m and a similar surface.

The measurements related to the itineraries of the roles and properties that attend the two play structures served as an a priori platform, thus systematizing the recording of motor behaviors through a system composed of four criteria and 15 categories. The follow-up of an observational methodology (Anguera and Hernández-Mendo, 2014, 2016) allowed us to delve into the scarce knowledge available regarding the behavior of players in the motor triad as a play scenario.

The final records were made by two experts in motor games. Previously, and to construct the recording tool, the observers analyzed images taken from different motor games, but developed under the same structures of the present study, with the purpose of agreeing on the degrees of freedom of the categories and suitable nuclear criteria. Once the tool was available, and using the software LINCE (Gabín et al., 2012), the images of the explained games were recorded. When the acceptable levels of reliability in the social sciences had been calculated, and checked, the definitive images were recorded using the same software.

The data quality control was carried out, in order to calculate the inter-observer and intra-observer reliability and validity. Pearson and Spearman correlation coefficients were used, as well as the theory of generalizability (Cronbach et al., 1972). Generalizability analysis was used to estimate accuracy, validity, reliability (Blanco-Villaseñor et al., 2014). In addition, the role sistem acts as a theoretical construct for the description of observed behaviors (Parlebas, 1981). The lowest values reached were 0.954 inter-operator per 0.964 intra-observer, Spearman coefficients in both cases. The JGRC/M model was used to calculate the variance (0%) attributable to the measurements made by each observer at two different times. Also, the JGRC/O model was used to calculate the inter-observer variance, repeating the procedure in the comparison made by both evaluators, obtaining values of 1%. The tools used to complete the data quality were the Generalizability Study GT program, v.2.0E (Ysewijn, 1996), the SAS statistical package (v.9.1.3) and the SPSS program (v.20).

### Data analysis

After transcribing video recording data was analyzed using Theme 6.0. Searching for temporal patterns default search parameters were used, except level of significance was set at 0.005 and minimum occurrences at 2.

## Results

**Figure 1:**
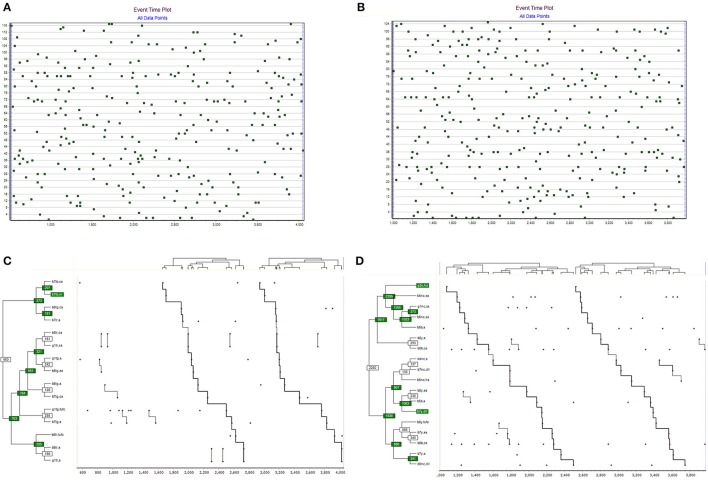
The event time plot **(A,B)** displays the distribution of registered events over time. Time is on the X-axis and event type number is on the Y-axis. Bottom on the left of the image, the most complex event time plot and T-pattern found in group 1 **(C)** when playing the triad game the “the maze” (modified). The same on the right of the image for group 2 **(D)**.

The two most complex T-patterns detected for groups 1 and 2 are displayed in Figure 1. The event time plot (top part of figure) illustrates the temporal distribution of transcribed events (the horizontal line representing the observation period and the vertical line occurrence time event types registered). For group 1, 117 different event types were registered, mounting to 278 data points (data rate 0.08). For group 2, 105 different event types were registered, mounting to 305 data points (data rate 0.10). The pattern statistics differed between the two groups. For group 1, 402 different patterns were detected, occurring 833 times. For group 2, 1,466 different patterns were detected, occurring 3,048 times.

The group 1 most complex pattern detected, displayed in Figure 1 (left side), consists of 15 different events, occurring with significantly similar time interval between themselves, on two occasions during the observation period. The pattern duration covers 61% of the observation period. The group 2 most complex pattern detected, displayed in Figure 1 (right side), consists of 18 different events, occurring with significantly similar time interval between themselves, on two occasions during the observation period. The pattern duration covers 89% of the observation period.

In the first game, specifically with group 1 composed of three teams (TR, TG, TB), a pattern tree graph with a greater verticality than in group 2 could be observed. The most dependent relations of group 1 were intragroup and not intergroup. The behavior of the TR team player (b5tr, ca) links its appearance in the game to the captive player (g1tr, ca). The TG player (g1tg, a) is imprisoned a prisoner and timely attached to the dodge action (b5tg, ea). The same player (b5tg, ea) is captured and related to the player's catch action (b7tg, ca).

The player of the TG team (g1tg, tufc) performs release actions related to fellows that are captured (b7tg, a). Finally, from the pattern tree graph, we find a group of three behaviors that link the release of partners (b6tr, tufc) to the existence of fellow prisoners (b5tr, a) and (g1tr, a), similar to the previous case.

In the second group of the same game, the teams (Y, NC, and B) showed through the pattern tree graph more intergroup behaviors with recurrent time dependence than intragroup ones. A prisoner of team Y (b6y, a) with a catcher of team B (b5b, ca). We observed a group of three behaviors of the NC team of the prisoner role in attention (b5nc, a) with displacements to free places (b7nc, dll) and fleeing (b5nc, ha). Also, (b5y, ea) and (b5b, a), like (b6y, tufa) with (b7y, ea) and (b5b, ca).

**Figure 2:**
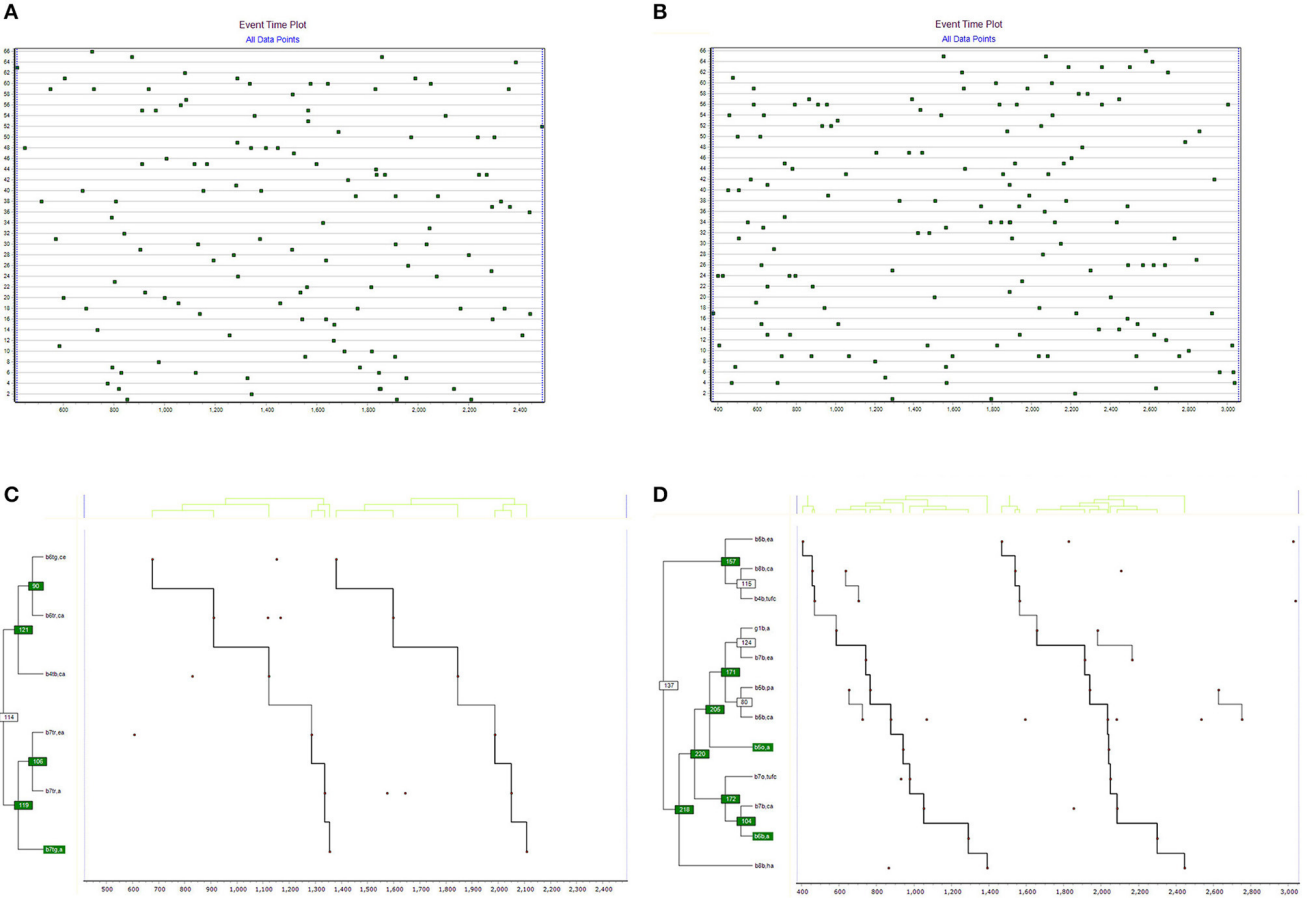
The event time plot **(A,B)** displays the distribution of registered events over time. Time is on the X-axis and event type number is on the Y-axis. Bottom on the left of the image, the most complex event time plot and T-pattern found in group 3 **(C)** when playing “the three fields” triadic game (modified). Also, on the right of the image for group 4 **(D)**.

The two most complex T-patterns detected for the groups 3 and 4 are displayed in Figure 2. The event time plot (top part of figure) illustrates the temporal distribution of transcribed events (the horizontal line representing the observation period and the vertical line occurrence time event types registered). For group 3, 25 different event types were registered, mounting to 66 data points (data rate 0.06). For group 4, different event types were registered, mounting to 66 data points (data rate 0.06). The pattern statistics differed between the two groups. For Group 3, 36 different patterns were detected, occurring 73 times. For group 4, 97 different patterns were detected, occurring 97 times.

The group 3 most complex pattern detected, displayed in Figure 2 (left side), consists of 6 different events, occurring with significantly similar time interval between themselves, on two occasions during the observation period. The pattern duration covers 68% of the observation period. The group 4 most complex pattern detected, displayed in Figure 2 (right side), consists of 11 different events, occurring with significantly similar time interval between themselves, on two occasions during the observation period. The pattern duration covers 73% of the observation period.

Group 4 shows a greater time dependence on game behaviors than group 3. In this group, the captive player of the TG team (b6tg, ce) caused captures (b6tr, ca) in the player belonging to the TR team. The two previous behaviors relate to the captures of a player of the TB team (b4tb, ca). Also, the dodging of the player belonging to the team TR (b7tr, ea) caused that same player to fall prisoner (b7tr, a). The two responses above were linked to the TG team reaction (b7tg, a).

The most obvious time recurrences were established with team B players. First, catches of team B (b8b, ca) with releases that another player from the same team performed (b4b, tufc). Also between the player (g1b, a) of the same team and (b7b, ea). Another linkage found corresponded to behaviors within team B and player (b5b, pa) and (b5b, ca). Other relationships that were part of the behavioral cluster, although less remarkable, would be those shown among the last four behaviors mentioned, to cause the identification of the behavior of team O (b5o, a) as a captured player. When team O freed teammates (b7o, tufc), actions were activated on team B to capture (b7b, ca), while prisoners on hold (b6b, a). Finally, the flight behavior of team B (b8b, ha) was activated.

## Discussion

A powerful data pattern detection technique was used, Theme 6.0 (Magnusson, 1996, 2000) to address the comparison of two triad structures of motor games to confirm or reject the existence of T-patterns in practice. The detection of T-patterns provides an accurate fusion of motor responses during the game to generate a time cluster with which to solve the triadic complexity; this fusion of motor responses emerged under the same system of roles for both games and it only limited the actions of the action structure of the triad.

The analysis of play structures (Pic and Navarro, 2017) in motor games, taking roles into account (Table 1), anticipated some key elements: (a) smaller relational limitations of structure 1 on structure 2; (b) increase of the antagonistic density of game 1 on game 2; (c) influence of the activation of structural properties in the two triad games and their consequences. This anticipated forecast theoretically requires to be contrasted with the results obtained. Therefore, we next assess the complexity of the T-patterns found in relation to the properties noticed in the structures, and their transformation from game roles to observed behaviors. In this way, advancing in the conciliation between play practice and a previous communication analysis tries to overcome the conjecture. In general, the analysis is based on ecological conditions (Araújo et al., 2006), facilitated by a methodology that allows the registration in natural environments (Anguera and Hernández-Mendo, 2016), and by the inclusion of a time dimension (Magnusson, 1996, 2000), in full harmony with the reality of the triadic events of motor practice (Pic and Navarro, 2017).

### Complexity of T-patterns and relational constraints on triad structures

One aspect of T-patterns is the complexity of patterns detected. The more structured behavioral phrases of the cluster detected by Theme (Jonsson et al., 2006) confirm the high relational complexity of the players when playing. Specifically, between the two groups practicing the same game the maze (modified) made a total of 1,868 T-patterns composed of 3,881 different behaviors, whereas in “the three fields” (modified) game they only reached 133 T-patterns composed of 170 ludic behaviors. Although it is true that the structural formula of “all against all” facilitated the relational exchange between participants, the motor response is put open to debate before the context influence (Araújo et al., 2014), subjecting players to comply with what is allowed by the rule within a given structure and under the organization of the role. In view of this structural condition, the results confirmed the existence of T-patterns in both triadic games. However, the time recurrence shown by the pattern tree graphs in the maze game (modified) reveals differences with respect to “the three fields” game (modified), joining the patterns found in other specific contexts (Fernandez et al., 2009; Lapresa et al., 2013a,b; Cavalera et al., 2015).

The origin of these differences refers to the nature of motor interaction (Parlebas, 1981) and to the intensity of antagonism (Heider, 1946; Table 1). In the maze (modified) we found that the space for collaboration was scarce (one collaborative behavior by six antagonists) compared to the three fields (modified), with one collaborative behavior by three antagonists. This extremely antagonistic scenario in of the maze game, requires many prisoner releases for the ludic system to be able to adapt (Passos et al., 2016). The facilitation of the capture as a priority objective to win needs more releases than in “the three fields” game (modification). In this sense, it is a less rigid formula and more adaptable to errors and successes of the players. The number of T-patterns reflects this high combinatorial complexity.

The search for T-patterns shared by both games or groups was unsuccessful and calls for an interpretation. Triadic structures are complex relational archetypes, subject to the emergence of written properties in the structure, but knowing these play systems does not guarantee their prediction. The differences between the four groups when playing two game structures showed different T-patterns. Perhaps because of the decision-making ability of the players, or maybe because of the restrictions of Theme to identify T-patterns with the subjects of the study labeled with event occurrences, or due to the vertiginously driven demands for situational adaptations.

Players may in many cases select automatic responses when facing the difficulty of having short time frames to react. On the other hand, it should not be forgotten that players do not have a decisive recipe (Araújo et al., 2014). Therefore, and in this sense, the decision is subject to great variation and, consequently, the groups and structures of the game do not explain by themselves the similarities in the obtained in the pattern tree graphs.

### Triadic T-patterns and emerging properties (circulation and reciprocity)

What keys have been activated in the games studied following the detection of T-Patterns? Reciprocity, when it is antagonistic (mirror-like, e.g., catcher-dodger), is determinant in the triad analysis because it hinders circulation fulfillment. That is to say, it is inversely proportional: to more reciprocity between the sum of existing duels, less circulation in one direction. This property justifies the structural paradox when the relation is fulfilled in a single sense, as happens in “the three fields” (modified). In this sense, it is a property of the circulation network, transformed into a triad constrains (Davids et al., 2008). It is thus that the lack of strategic organization detected by Theme in “the three fields” (modified) is not accidental but causal, and it was more disorganized. In “the maze” there was a greater decisional alternative (Araújo et al., 2006) to address the problems that arose from the game.

If we focus on the salvator role, on it rests essentially the continuity of the game, in a systemic sense, but it affected each group in a different way. In group 1, the TUFC record was identified by THEME as T-pattern. However, group 2 practicing the same game (the maze) was found to be TUFA (action to free opponents of the rival team, developed by rescuers). According to this, the need for releases in the maze mentioned in the previous section seems to be reinforced due to the communication structure (Table 1), but also to the specificity of each group, since it was only in the second of them that Theme identified it.

Specifically, in the last relational framework identified by Theme, in the second group of “the maze” game, which indicates that the team would have been in a difficult situation, also supported because the rival team could make effective captures (b5b, ca), is the release of opponents made by the player (b6y, tufa) linked to the player of the same team when performing dodges (b7y, ea). It may be this criticality what leads the teams to partnership with rivals. Based on the above, its positive or negative good value (Heider, 1946) is vital to understand the strategic specificity of each group and the systemic need for liberation for both groups during play.

We have already alluded to a smaller elaboration of the T-patterns in the three fields in front of the game the maze, backed by the number of T-patterns but also by a greater strategic and temporary structuring of the motor action. The collaborations or release actions between players in the first game showed different behaviors regarding the previous game. Release actions among fellow players (TUFC) were only identified in group 4. That is, the lower number of T-patterns identified in the three fields affected the savior role. In group 3, there was no collaboration to make releases between fellow players or opponents, which could indicate a state of normative incomprehension, culturally understandable in the players for a lack of triadic experiences that group 4 put into practice. The property of circulation and its paradoxical effects on the motor decision, could explain the high strategic disorganization identified by THEME in the third triad group. Again, structure and play roles described the pattern of the motor decision, showing the situational demands that were brought by the groups and found by the relevant detection of temporary recurrences in view of previous studies in sports (Jonsson et al., 2006).

In conclusion, T-patterns have solved the underlying complexity of the two different game structures and their groups, beyond the systemic solution, showing how players are temporarily confronted in natural contexts of practice.

## Conclusions

This study has demonstrated the advantages of using a technique pattern detection to address the internal complexity of the motor triad. The inclusion of a time dimension has meant an advance for the interpretation and analysis of data from ecological contexts, confirming the properties of the ludic structure.

The decisional complexity of the T-Patterns was different in “the maze” and “the three fields” triadic games. It was also reinforced by the number and composition of T-patterns. The relational strategies identified in the four groups were different, since no similarities were found; which confirms the high complexity of each game developed by each group. This high complexity shows a specific variability for each triadic motor game.

## Limitations

Among the limitations that accompanied the study, the inclusion of the spatial criterion as a facet of it would be worth mentioning. Similar studies could replicate this research, with different populations and age groups, to have a thorough knowledge about the triadic effects. Performing analysis aimed at detecting the inhibition and activation of particular play behaviors would add an important explanatory value. The theory of motor play needs to put on hold the circle of appreciation and move on to research, in order to know what these and other motor games hide, as exceptional formulas of social interaction.

## Author contributions

MA and VN-A have contributed to the theorical and methodological development of the article while GJ has contributed with the data analysis. Results and discussion have been prepared by all authors.

### Conflict of interest statement

The authors declare that the research was conducted in the absence of any commercial or financial relationships that could be construed as a potential conflict of interest.
